# Predicting the Intention and Adoption of Near Field Communication Mobile Payment

**DOI:** 10.3389/fpsyg.2022.870793

**Published:** 2022-04-08

**Authors:** Chinnasamy Agamudainambi Malarvizhi, Abdullah Al Mamun, Sreenivasan Jayashree, Farzana Naznen, Tanvir Abir

**Affiliations:** ^1^Faculty of Management, Multimedia University, Cyberjaya, Malaysia; ^2^UKM – Graduate School of Business, Universiti Kebangsaan Malaysia, Bangi, Malaysia; ^3^UCSI Graduate Business School, UCSI University, Kuala Lumpur, Malaysia; ^4^Faculty of Business and Entrepreneurship, Daffodil International University, Dhaka, Bangladesh

**Keywords:** UTAUT2, NFC M-payments, perceived risk, intention to adopt, youth, Malaysia

## Abstract

With the increasing use of mobile devices and new technologies, electronic payments, such as near field communication (NFC) mobile payments, are gaining traction and gradually replacing the currency-based cash payment methods. Despite multiple initiatives by various parties to encourage mobile payments, adoption rates in developing countries have remained low. The purpose of this research is to explore the prime determinants of NFC mobile-payment adoption intention and to develop a model of mobile payment adoption that includes perceived risk (PR) as one of the major elements by extending the UTAUT2 theory components. An online survey was used to acquire data from 370 NFC mobile payments users for the current study. To validate the components and their correlations, structural equation modelling (SEM) was implemented. According to the findings, performance expectancy (PE), hedonic motivation (HM), social influence (SI), and facilitating conditions (FC) have substantial impacts on the consumers’ intentions to adopt NFC mobile payments (INFC). Effort expectancy (EE) and PR were reported to have no considerable effects on the adoption intention. In addition, INFC is revealed to be a major mediator between the associations of the actual adoption of NFC mobile payment (ANFC) with PE, HM, and SI. The findings of the study would assist providers and marketers in better understanding of the consumers’ behavior, designing effective marketing strategies to enhance the consumers’ positive intentions, and achieving the mass adoption of NFC mobile payments in different environmental contexts.

## Introduction

Mobile technology has become an essential component of daily life that enhances socio-economic growth (Baganzi and Lau, 2017). The increased adoption of smartphones, mobile devices, and numerous e-commerce platforms contributes to the rapid expansion of mobile payments worldwide. One of the most novel and innovative technological advancements in the sector of banking and finance is the mobile payment system. This feature allows users to effortlessly make financial dealings (e.g., fund transfers, bill payments, and balance inquiries) *via* mobile devices that are independent of time and place (Alalwan et al., 2017). Notably, mobile payment services have become critical and indisputable financial technology services over the last decade due to their increasing prevalence *via* smartphones and other portable devices, including the facilitation of financial transactions *via* the newest and most advanced technology channels.

Near field communication (NFC)-based mobile payment is one of the newest mobile payment technologies that are growing in popularity (Khalilzadeh et al., 2017). Portable devices are coupled with NFC tags, which may function as digital wallets and allow customers to seamlessly perform transactions by waving their devices over NFC-furnished payment terminals and point-of-sale (POS) (Gerstner, 2015). The wide range of NFC-based transaction applications, such as in-store purchases, public transportation tickers, and paying bills, are employed in all payment terminals integrated with a dedicated contactless connectivity processor, remarkable convenience in use, and improved security features (Liébana-Cabanillas et al., 2021). Despite the convenience of NFC M-payments and their wide range of benefits, the adoption of these payments among Malaysians remains in the early stages with a lower adoption rate compared to other nations across the world (Tan et al., 2014).

According to a World Bank report in 2018, 3.8 billion adult individuals worldwide (69% of the total adult) currently own bank accounts or accounts with mobile money service providers (World Bank Report, 2018). According to Mckinsey, the decrease in the global cash transactions occurs daily, which was proven through Sweden (9%) with the lowest percentage of cash being used in total transactions by volume at the end of 2020. This was followed by the Netherlands (14%) and the United Kingdom (23%) (McKinsey, 2020). With the establishment of Apple-Pay in 2014, significant growth in NFC M-payments usage has been identified in recent years, with more merchants currently processing mobile payments (Khalilzadeh et al., 2017). Furthermore, Malaysia’s government and the Central Bank of Malaysia (Bank Negara Malaysia) are actively promoting the usage of non-cash payments by reducing the fees for e-payments to encourage enterprises and customers toward the use of cashless payment methods (Moghavvemi et al., 2021). In July 2016, Maybank Malaysia initiated the Maybank-Pay, which was considered the first mobile-wallet payment application in Malaysia, to encourage cashless payments *via* smartphones. Maybank-Pay, which allows cardholders to store card credentials on their mobile devices, was designed to transform electronic payments in Malaysia (Maybank Press, 2016). Despite the numerous efforts of the government and financial institutes to promote mobile payment in Malaysia, perceptions and adoption rates remained relatively low, while Malaysia remained a cash-based society with 72% of transactions taking place in cash (McKinsey, 2020). Although Samsung was actively working in collaboration with Malaysian banks (e.g., CIMB, Hong Leong Bank, Standard Chartered Bank, and Citibank) in February 2017 to activate Samsung-Pay and establish the dynamic mobile-payment systems and effortless transaction methods for the consumers of their smart devices, empirical evidence has proven that only a small percentage of Malaysians was using Samsung-Pay (Moghavvemi et al., 2021). Different communities may have diverse cashless payment methods and functionalities, which would result in a diverse outcome of adoption due to consumers’ behavioral and technological post-adoption characteristics (Ariffin et al., 2021). Moreover, it has been suggested that numerous aspects relating to each country’s culture or diversities may also impact the user’s inclination to use mobile payment services which need to be investigated more rigorously (Flavián et al., 2020). Therefore, it is crucial for service providers to determine the factors that commonly motivate users to use NFC M-payments. To address the current gaps in practical usage scenarios, the aim of the current study is, therefore, to empirically explore and identify the most important determinants contributing to adoption intention and actual usage of NFC mobile payments among Malaysians.

Perceived ease of use, compatibility, perceived usefulness, social influence (SI), trust, attitude, and other factors have all been investigated widely in recent studies of mobile payments (Flavián et al., 2020). However, different results were gained due to the variances in sample selection, respondents’ demographic characteristics, and other sociological and economic circumstances (Hussain et al., 2019). Besides, Flavián et al. (2022) recommended that the user’s negative perceptions about technological insecurity and anxiety regarding automated financial systems (especially among the less experienced users) necessitate further rigor investigation. Hence, this study included perceived risk (PR) as an additional component expanding the UTAUT2 model, which has been studied less frequently, particularly in the context of NFC mobile payments in Malaysia. Taking these gaps and recommendations into account, the current study implemented UTAUT2 model to assess the theory’s applicability with this extended construct in the context of NFC M-payments adoption in Malaysia. Notably, the findings of the study will help academics to better understand the impacts of contextual factors on the consumers’ inclinations to embrace new financial technology. The insights would also assist fintech service providers to effectively develop their innovative service offerings and apply effective tactics to increase the customers’ willingness to embrace NFC M-payments. The outcomes of the current study provide customers behavioral analysis and accordingly would assist with business plans and recommendations for improving potential consumers’ intention to use NFC M-payments through distinct marketing approaches.

## Literature Review

### Theoretical Background

The unified theory of acceptance and the use of technology-2 (UTAUT2) is developed by extending the UTAUT theory, which provides an integrated view by excluding the concurrency and iterations of similar elements and integrating the constructs from eight popular theories and models, i.e., theory of reasoned action (TRA), technology acceptance model (TAM), innovation diffusion theory (IDT), theory of planned behaviour (TPB), and social cognitive theory (Hussain et al., 2019). Three additional factors (price value, hedonic motivation, and habit) were added with four original elements of the UTAUT theory (e.g., performance expectancy [PE], effort expectancy [EE], SI, and facilitating condition [FC]), which resulted in the establishment of the UTAUT2 (Venkatesh et al., 2012). Compared to the original UTAUT, the expansion included in the UTAUT2 contributed to a considerable improvement in the variance, as indicated through the consumers’ intentions to adopt (56–74%) and actual usage behavior (40–52%) (Tandon et al., 2016). Specifically, the UTAUT2 has been proven to be highly efficient at interpreting mobilepayment technology adoption intentions and actual usage behavior among users (Gupta and Arora, 2019). Moreover, a considerable number of earlier studies employed the UTAUT2 to assess the factors that contributed to the adoption of NFC M-payments (Slade et al., 2015; Morosan and DeFranco, 2016; Khalilzadeh et al., 2017; Karjaluoto et al., 2020; Liébana-Cabanillas et al., 2021). Given that it is a widely used theory in addressing both external and internal drivers of any new technology adoption (Koenig-Lewis et al., 2015). This study adopted the UTAUT2 model as a theoretical underpinning.

Numerous earlier studies (Morosan and DeFranco, 2016; Oliveira et al., 2016; Gupta and Arora, 2019; Hussain et al., 2019) found that the price value was not a significant indicator of adoption intention behavior in the context of upgraded technology in contactless payments. Based on literature findings and arguments, price value was not considered a factor in this study. To explore the impact of habit, users require a high level of experience and continuous usage records after the adoption of new technology (Alalwan et al., 2017). Furthermore, a new technology that has not gained widespread market acceptability may be faced with obstacles in building a habit among users (Oliveira et al., 2016; Lin et al., 2020). Considering the fact that many of the participants in this study were not using the NFC M-payments on a regular basis, the habit was not considered a determinant of users’ intention to adopt the NFC M-payments.

The expansion of the UTAUT2 model has been suggested through the incorporation of other determinants associated with regions and cultures (Sobti, 2019). To illustrate, researchers (Khalilzadeh et al., 2017; Karjaluoto et al., 2020; Liébana-Cabanillas et al., 2021; Penney et al., 2021) modified the UTAUT2 through PR as a determinant that strongly encourages the mobile payment adoption. Following previous researchers’ recommendations, this study extended the UTAUT2 model by including PR along with the other determinants.

### Development of Hypotheses

#### Performance Expectancy

Consumers tend to be more likely to purchase new technologies when the technologies are seen to be advantageous and capable of improving their daily performance (Venkatesh et al., 2003). Mobile payment’s innovativeness, as well as its effectiveness as a viable alternative to traditional payment methods, increases the users’ perceived usefulness when they see how it differs in a friendly manner, in a more convenient and advantageous way than other methods (Flavián et al., 2020). The perceived likelihood that technology would transform the way a customer accomplishes his objective is established as PE and it was revealed to enhance the attitude and adopting intention of NFC M-payments (Liébana-Cabanillas et al., 2017). In regards to mobile payments, performance expectancy (PE) could be measured through the extent to which customers perceive that mobile payments would improve their transaction performance with simplicity, convenience in payments, timeliness, service effectiveness, and fast responses (Penney et al., 2021). Furthermore, Narteh et al. (2017) argued that individuals utilize M-payments further upon realizing the advantage and convenience. The PE was proven in previous studies to be strongly associated with the customers’ inclination to use the NFC M-payments (Slade et al., 2015; Morosan and DeFranco, 2016; Karjaluoto et al., 2020; Liébana-Cabanillas et al., 2021). Accordingly, the following hypothesis was proposed based on the evidence from existing literature:

H1: *Performance expectancy positively affects the adoption intention of NFC M-payments.*

#### Effort Expectancy

Effort expectancy denotes the degree to which technology features are simple to use and understandable due to their relation to customer adaptation (Venkatesh et al., 2012). This definition implies that the little effort is required by users to increase the likelihood of adoption of any technology (Narteh et al., 2017). As a result, EE is regarded as one of the most significant factors in the preference to embrace new technology (Liébana-Cabanillas et al., 2017). In the context of mobile payments, EE refers to the ease with which users may sign up for services and make transactions with the amount of steps (Penney et al., 2021). Furthermore, the perception of a lack of control or skill in dealing with technology might lead to the rejection of new solutions (Flavián et al., 2022). The particular characteristics of mobile-banking technologies necessitate a certain level of expertise, competence, and understanding, whereas EE may play a critical role in influencing the customers’ intention to accept the technology by easing the procedures in operating it (Alalwan et al., 2017). In terms of mobile payments, when a user is more aware of his or her requirements and understands how technologies may well be handled, the user’s capacity to evaluate the ease of the use of technology improves (Flavián et al., 2020). It was found in numerous earlier studies that the EE was an important influential determinant in shaping the consumers’ intentions to embrace mobile payments (Gupta and Arora, 2019; Hussain et al., 2019; Karjaluoto et al., 2020). Based on the evidence from the literature, the following hypothesis was suggested:

H2: *Effort expectancy positively influences the adoption intention of NFC M-payments.*

#### Hedonic Motivation

Hedonic motivation denotes the feelings of pleasure and joy gained from the adoption of any new technologies (Brown and Venkatesh, 2005). The concept of HM encompasses intrinsic benefits, such as enjoyment, satisfaction, joyfulness, and entertainment, in the use of new technology (Venkatesh et al., 2012). However, consumers may embrace new technologies not only as equipment or services to improve the quality of life, but also as sources of enjoyment (Koenig-Lewis et al., 2015). In respect of e-wallet technologies, service providers might provide many benefits, such as discounts and cashback, which can boost customer pleasure and result in wide reuse intention (Ariffin et al., 2021). Hussain et al. (2019) described HM as a delightful experience in which individuals feel satisfied upon transferring a proportion of their earnings to their family members *via* the mobile payment service and paying for their family’s fundamental needs despite residing at different places. Moreover, the enjoyment or pleasure gained from adopting new technology has a significant contribution in enhancing the consumers’ usage intention (Alalwan et al., 2015). In numerous earlier studies, HM was found to be positively and substantially associated with mobile-payment adoption intention (Morosan and DeFranco, 2016; Khalilzadeh et al., 2017; Lin et al., 2020). Subsequently, all these arguments and evidence led to the development of the following hypothesis:

***Hypothesis 3 (H3):*** *Hedonic motivation positively influences the adoption intention of NFC M-payments.*

#### Perceived Risk

The degree of assumed uncertainty among users regarding the threats of deploying a certain technology is referred to as PR (Tan and Lau, 2016). PRs rise in lockstep with the level of any uncertainties while using any products or services (Abbasi et al., 2021). Furthermore, users have the strongest apprehension regarding privacy invasions, loss of personal data, and hacked transaction data upon performing NFC M-payments (Liébana-Cabanillas et al., 2021). The predicted risks in the studies could comprise any unacceptable results for clients, such as money loss, psychological anxiety, long waiting time, and lengthy process (Baganzi and Lau, 2017; Narteh et al., 2017). Concerns about security risk decrease the consumers’ intention to embrace automated fintech services (Flavián et al., 2022). Many studies in various cashless payment contexts (Khalilzadeh et al., 2017; Karjaluoto et al., 2020; Liébana-Cabanillas et al., 2021; Penney et al., 2021) established that the PR is negatively associated with the adoption intention of mobile payment systems. In terms of robo-fintech services, Flavián et al. (2022) empirically revealed that the experienced users should be less agitated by technology threats that may increase their adoption intention. Based on the aforementioned descriptions, the following hypothesis was suggested:

***Hypothesis 4 (H4):*** *Perceived risk negatively influences the adoption intention of NFC M-payments.*

#### Social Influence

Social influence has been defined as the degree of importance in adopting new technology for individuals (Venkatesh et al., 2003). The SI determines the effects of external influences on the users’ behavioral intention, which involves the opinions from the family, friends, relatives, and superiors regarding the adoption of technology (Baptista and Oliveira, 2015). Simultaneously, the degree of the users’ readiness to reconsider opinions should have a substantial influence on their adoption of mobile payments (Narteh et al., 2017). Customers gain more knowledge while they adopt new technologies, which will raise awareness amongst their peers through word-of-mouth and recommendations (Flavián et al., 2022). SI has been empirically demonstrated to have a positive and significant impact on the users’ pleasure to use the e-wallets (Ariffin et al., 2021). As a substantial predictor of adoption intention among users, SI was supported in previous research and established association among customers’ inclination to adopt various cashless payment methods, such as NFC M-payments (Morosan and DeFranco, 2016; Khalilzadeh et al., 2017; Liébana-Cabanillas et al., 2021), mobile-payment (Oliveira et al., 2016; Hussain et al., 2019), e-wallet (Ariffin et al., 2021), and mobile-money (Narteh et al., 2017; Penney et al., 2021). Based on the findings on the literature, the following hypothesis was proposed:

H5: *Social influence positively affects the adoption intention behavior of the NFC M-payments.*

#### Facilitating Conditions

Facilitating conditions are identified as the views of customers regarding the availability of resources and support to accomplish any task that employs new technologies (Venkatesh et al., 2012). Ability, time, and resources all play a significant role in anticipating a person’s intention to engage in behavior (Abbasi et al., 2021). Mobile payment mandates that users gain several fundamental skills and guidelines, such as the ability to use Mobile payment apps and send–receive text messages that are regarded as FCs (Kiconco et al., 2020). The FCs, which include the availability of mobile payment agents, merchants, and service accessibility on mobile devices equipped with essential features and software, would improve the efficiency in usage of the technology (Penney et al., 2021). Moreover, financial resources, technological assistance, user guidelines, and well-constructed infrastructure are the external FCs that could impact the likelihood of adopting any new technology among consumers (Yang et al., 2021). Consumers may become more inclined to adopt mobile-banking technologies when they obtain access to standard service support and resources (Alalwan et al., 2017). Additionally, FCs were found to have noteworthy positive influences on the adoption of mobile payment technologies (Koenig-Lewis et al., 2015; Chawla and Joshi, 2019; Lin et al., 2020). Based on the previous research findings, the following hypothesis was suggested:

H6: *Facilitating conditions positively influence the adoption intention of NFC M-payments.*

#### Intention to Adopt and Actual Adoption of Near Field Communication M-Payments

The UTAUT is based on the belief that the intentions to adopt new technology affects actual adoption performance (Venkatesh et al., 2012). Mobile payment is regarded as a disruptive technology (Schmidthuber et al., 2018), and hence attracts the attention of marketing researchers (Flavián et al., 2020). The intention is a predecessor to real usage behavior as it represents an individual’s willingness to engage in a given activity (Sivathanu, 2019). The users with higher behavioral intentions to embrace new technologies have a higher inclination to be the ultimate users (Leong et al., 2013). According to previous discussion and literature analysis, PE, EE, SI, HM, PR, and FC are the major components that affect users’ adoption intention of NFC M-payment, which may vary depending on country and cultural context, technological environment, and users’ demographic context (Liébana-Cabanillas et al., 2017). Moreover, some users tend to embrace a technology when it is still in its early life, whilst others prefer to wait for it to flourish before making a decision to adopt it (Pal et al., 2015). It has been observed that consumers that are more receptive to new technologies have the highest likelihood of becoming early adopters (Oliveira et al., 2016). According to earlier study findings, the ultimate adoption of mobile payment technology was highly dependent on the users’ inclination to adopt the technology (Koenig-Lewis et al., 2015; Oliveira et al., 2016; Gupta and Arora, 2019; Karjaluoto et al., 2020). This association was supported by the following hypothesis:

H7: *Intention to use NFC M-payments positively impacts the actual adoption of NFC M-payments.*

## Research Methodology

### Population Sample and Data Collection

To evaluate the adoption intention and actual acquisition of NFC M-payments among Malaysian youth, a cross-sectional research methodology and quantitative technique were employed in this study. People who aged from 18 to 40 years old were categorized as youth and working adults by the International Labor Organization (International Labor Office [ILO], 2013). According to the most recent official census in 2020, 12.1 million Malaysians who aged 15–40 years old represented 37.12% of the country total population (Department of Statistics Malaysia [DOSM], 2020). Moreover, convenience sampling was employed to gather the most accessible respondents. With the use of G-Power version 3.1.9, the size of the sample for this study was determined to be 153 through an effect size of 0.15 and power of 0.95 for seven determinants (Faul et al., 2007). Nevertheless, data were obtained from 370 Malaysian youth to manage any potential problems caused by the small sample size. Following that, the online survey was conducted through a Google Form created with the study questionnaire. The online questionnaire was distributed to respondents through emails and social media (e.g., Facebook and WhatsApp).

### Research Instrument

With minimal modifications, the survey questionnaire was derived from previously tested and verified surveys conducted in other studies. The questionnaire was structured using a clear and unbiased method to ensure that participants would be able to understand and answer the questionnaire without any ambiguity. Furthermore, to measure PE six items were adopted from Wei et al. (2021) and Karjaluoto et al. (2020), while the EE consisted of six items, which were adopted from Venkatesh et al. (2003), Karjaluoto et al. (2020), and Wei et al. (2021). The five items that gauged HM were implemented by Karjaluoto et al. (2020) and Liébana-Cabanillas et al. (2021). The PR was assessed using five items adopted from Karjaluoto et al. (2020) and Sobti (2019). For the measurement of SI, five items from Venkatesh et al. (2003), Rahi and Ghani (2019); Dutot (2015), and Wei et al. (2021) were consolidated. The FC was measured with five items taken from Morosan and DeFranco (2016) and Wei et al. (2021). Moreover, six items to evaluate INFC were acquired from Dutot (2015). Finally, four items for measuring ANFC were obtained from Morosan and DeFranco (2016). Overall, all the items were requested to be answered through a five-point Likert scale, in which its five items ranged from “strongly disagree” to “strongly agree.”

### Common Method Variance

Harman’s single-factor test is the most often used strategy among academics to manage Common Method Variance (CMV), given that this test affirms that CMV has no impact on the study model (Chang et al., 2010). In this study, the single-factor test resulted in the highest factor accounting for 48.974%, which was lower than the recommended maximum limit of 50%. This result also indicated the insignificant effect of CMV on this study (Podsakoff et al., 2012).

### Multivariate Normality

Although the multivariate normal distribution is not necessary for the PLS approach, Peng et al. (2012) cautioned against making broad generalizations about PLS capacity for evaluating a model, given that this action might violate the criteria of multivariate normality. The multivariate data normality for this study dataset was evaluated using Web Power, which was an online tool for validating data normality.^1^ Upon the assessment of the multivariate coefficient (output of skewness and kurtosis computation), *p*-values were found to be below the recommended threshold value of 0.05 (Cain et al., 2017), proving that the dataset was not normal.

### Method of Data Analysis

Given that this study was an exploratory type with non-normality issues, variance-based partial least squares structural equation modelling (PLS-SEM) was employed to analyse the study dataset. The PLS-SEM is a causal modeling approach that minimizes the explained variance of endogenous latent components (Hair et al., 2011). As highlighted by Hair et al. (2019), the data analysis comprises (a) descriptive analysis (mean and standard deviation [*SD*]), (b) internal reliability (Cronbach’s alpha and composite reliability [CR]), (c) convergent validity using average variance extracted (AVE), (e) discriminant validity (Fornell–Larcker criterion, and loadings and cross-loadings), (f) path coefficient (β), (g) predictive relevance (*Q*^2^), and (h) coefficient of determination (*r*^2^).

## Data Analysis

### Demographic Characteristics

Table 1 illustrates the demographic characteristics of the participants. The majority of the 370 respondents (63.5%) were male. Following that, 60.0% of the participants were aged between 21 and 25 years old, while 18.9% of them were under the age of 21, and 29.1% of them were aged between 26 and 30 years old. Furthermore, the monthly income under RM2,500 was the most common amount among the participants (67.6%), followed by the monthly income between RM2,501 and RM5,000 (23.8%). While only 10.8% of the respondents were married, 88.9% of them were single. The majority of participants (65.7%) had completed Bachelor’s degree or equivalent, while 17.6% of them had fulfilled diploma/technical school level education. Moreover, 89.7% of respondents stated that they reside in cities, while 10.3% of them stated that they reside in rural regions.

**TABLE 1:** Demographic characteristics.

Item	N	%	Item	N	%
**Gender**			**Marital Status**		
Male	235	63.5	Single	329	88.9
Female	135	36.5	Married	40	10.8
Total	370	100.0	Divorced	1	0.3
			Total	370	100.0
**Age Group**			**Education**		
Below 21 years	70	18.9	Secondary school certificate	33	8.9
21–25 years	222	60.0	Diploma/technical school certificate	65	17.6
26–30 years	78	29.1	Bachelor degree or equivalent	243	65.7
Total	370	100.0	Master’s degree	28	7.6
			Doctoral degree	1	0.3
			Total	370	100.0
**Average Monthly (RM)**			**Living Area**		
Below RM2500	250	67.6	Urban	332	89.7
RM2501-RM5000	88	23.8	Rural	38	10.3
RM5001-RM7500	13	3.5	Total	370	100.0
RM7501-RM10,000	8	2.2			
RM10,001-RM12500	5	1.4			
More than RM12500	6	1.6			
Total	370	100.0			

### Validity and Reliability

Cronbach’s alpha provides a reliability analysis based on the inter-correlations between the indicators. As demonstrated in Table 2, all of the indicators showed Cronbach’s alpha values of higher than 0.9, indicating that they are dependable. Furthermore, with a cut-off value of higher than 0.7, CR is employed as an alternative to analyse internal consistency (Hair et al., 2019). The CR values for all the components remained higher than 0.842 as shown in Table 2, indicating a high degree of internal consistency and reliability. Moreover, the AVE is a standard indicator for assessing convergent validity, which determines to what extent can the variation in the indicators be explained by the latent variables. All constructs with the AVE values of higher than 0.654 (Table 2) have proven a strong convergent validity due to their ability to explain over 65.4% of the variance in their indicators on average (Hair et al., 2017).

**TABLE 2:** Validity and reliability.

Constructs	No. items	Mean	Standard deviation	Cronbach’s Alpha	Dijkstra-Hensele’s *rho*	Composite reliability	Average variance extracted
Performance expectancy	6	4.108	0.825	0.926	0.926	0.942	0.730
Effort expectancy	6	4.157	0.780	0.945	0.946	0.956	0.784
Hedonic motivation	5	3.853	0.903	0.947	0.947	0.959	0.824
Perceived risk	5	3.303	0.827	0.801	0.793	0.842	0.517
Social influence	5	3.647	0.827	0.867	0.871	0.904	0.654
Facilitating conditions	5	3.931	0.784	0.909	0.910	0.932	0.734
Intention to adopt NFC M-payments	6	3.914	0.871	0.946	0.947	0.957	0.788

The Fornell–Larcker criteria are evaluated by emphasizing that the square root of the AVE of each predictor should have a greater value than the highest association with any other construct (Hair et al., 2017). As shown in Table 3, all of the elements fulfilled the Fornell–Larcker criteria required parameters. Additionally in Table 3, the Heterotrait–Monotrait Ratio (HTMT) values of all factors were reported below the threshold of 0.9 as recommended by Henseler et al. (2015). Cross-loading analyses were also performed to determine the discriminant validity. To assess the model appropriateness, an indicator’s outer loading on the associated construct should be more significant than any of its cross-loadings (e.g., correlation) on other constructs (Hair et al., 2017). The cross-loading values presented in Table 4 illustrated that all of the items fulfilled the criteria through their maximum loading value with respective components.

**TABLE 3:** Discriminant validity of constructs.

	PE	EE	HM	PR	SI	FC	INFC	ANFC
**Fornell–Larcker criteria**								
PE	0.854							
EE	0.817	0.885						
HM	0.729	0.743	0.908					
PR	0.207	0.228	0.346	0.719				
SI	0.574	0.565	0.628	0.514	0.809			
FC	0.661	0.721	0.645	0.378	0.703	0.857		
INFC	0.783	0.738	0.767	0.342	0.715	0.784	0.887	
ANFC	0.512	0.535	0.539	0.280	0.558	0.669	0.701	0.846
**Heterotrait-Monotrait Ratio (HTMT)**								
PE								
EE	0.874							
HM	0.778	0.786						
PR	0.200	0.196	0.309					
SI	0.634	0.621	0.690	0.527				
FC	0.721	0.778	0.695	0.362	0.787			
INFC	0.837	0.780	0.810	0.290	0.783	0.845		
ANFC	0.560	0.580	0.584	0.273	0.626	0.739	0.758	

*PE, performance expectancy; SI, social influence; EE, effort expectancy; FC, facilitating conditions; HM, hedonic motivation; PR, perceived risk; INFC, intention to adopt NFC M-payments; ANFC, adoption of NFC M-payments.*

**TABLE 4:** Loading and cross-loading.

Code	PE	EE	HM	PR	SI	FC	INFC	ANFC
PE1	***0.891***	0.725	0.595	0.125	0.471	0.590	0.667	0.428
PE2	***0.852***	0.682	0.680	0.196	0.552	0.580	0.681	0.447
PE3	***0.885***	0.725	0.604	0.198	0.507	0.564	0.693	0.443
PE4	***0.861***	0.676	0.659	0.268	0.518	0.565	0.674	0.452
PE5	***0.809***	0.682	0.563	0.116	0.403	0.544	0.639	0.430
PE6	***0.825***	0.698	0.631	0.153	0.485	0.542	0.656	0.425
EE1	0.759	***0.869***	0.612	0.175	0.457	0.625	0.621	0.439
EE2	0.757	***0.910***	0.682	0.175	0.496	0.680	0.690	0.472
EE3	0.750	***0.925***	0.663	0.197	0.515	0.658	0.686	0.495
EE4	0.727	***0.877***	0.666	0.196	0.507	0.635	0.656	0.469
EE5	0.713	***0.905***	0.664	0.191	0.496	0.629	0.651	0.494
EE6	0.633	***0.822***	0.660	0.285	0.533	0.599	0.614	0.474
HM1	0.677	0.705	***0.912***	0.288	0.562	0.591	0.696	0.486
HM2	0.696	0.709	***0.915***	0.306	0.537	0.606	0.724	0.519
HM3	0.650	0.668	***0.925***	0.309	0.601	0.594	0.685	0.474
HM4	0.658	0.651	***0.921***	0.350	0.579	0.572	0.688	0.489
HM5	0.625	0.639	***0.866***	0.316	0.574	0.564	0.687	0.477
PR1	0.219	0.227	0.364	***0.768***	0.361	0.315	0.302	0.258
PR2	−0.039	−0.002	0.128	***0.744***	0.264	0.185	0.101	0.131
PR3	−0.053	−0.031	0.144	***0.729***	0.242	0.113	0.070	0.103
PR4	0.210	0.233	0.269	***0.709***	0.486	0.320	0.327	0.217
PR5	0.096	0.090	0.099	***0.638***	0.265	0.218	0.136	0.150
SI1	0.377	0.393	0.475	0.519	***0.806***	0.491	0.506	0.363
SI2	0.407	0.421	0.459	0.406	***0.844***	0.506	0.537	0.412
SI3	0.505	0.485	0.452	0.383	***0.725***	0.615	0.572	0.456
SI4	0.539	0.516	0.523	0.362	***0.856***	0.630	0.648	0.516
SI5	0.468	0.452	0.615	0.425	***0.807***	0.580	0.604	0.487
FC1	0.532	0.599	0.552	0.313	0.594	***0.878***	0.650	0.583
FC2	0.616	0.689	0.602	0.323	0.596	***0.867***	0.704	0.580
FC3	0.625	0.685	0.594	0.331	0.648	***0.858***	0.737	0.556
FC4	0.513	0.549	0.488	0.344	0.619	***0.835***	0.589	0.557
FC5	0.544	0.566	0.527	0.311	0.558	***0.844***	0.678	0.589
INFC1	0.652	0.600	0.656	0.345	0.616	0.650	***0.851***	0.570
INFC2	0.702	0.663	0.710	0.316	0.670	0.712	***0.905***	0.643
INFC3	0.693	0.650	0.661	0.311	0.654	0.704	***0.888***	0.607
INFC4	0.697	0.648	0.672	0.282	0.601	0.680	***0.882***	0.629
INFC5	0.689	0.673	0.681	0.298	0.649	0.728	***0.903***	0.643
INFC6	0.734	0.694	0.701	0.276	0.614	0.698	***0.896***	0.637
ANFC1	0.377	0.373	0.443	0.259	0.475	0.535	0.540	***0.809***
ANFC2	0.466	0.467	0.466	0.196	0.444	0.574	0.612	***0.828***
ANFC3	0.439	0.459	0.457	0.264	0.476	0.568	0.615	***0.868***
ANFC4	0.445	0.452	0.424	0.237	0.471	0.587	0.603	***0.871***
ANFC5	0.435	0.506	0.489	0.233	0.496	0.565	0.589	***0.851***

*PE, performance expectancy; SI, social influence; EE, effort expectancy; FC, facilitating conditions; HM, hedonic motivation; PR, perceived risk; INFC, intention to adopt NFC M-payments; ANFC, adoption of NFC M-payments. Bold values are item loading, other values are cross-loading.*

### Path Analysis

The first section of Table 5 presents the analysis of the correlations between INFC and PE, EE, HM, PR, and SI, which demonstrated that PE (β-value = 0.348, and *p*-value = 0.000), HM (β-value = 0.251, and *p*-value = 0.000), and SI (β-value = 0.298, and *p*-value = 0.000) showed positive beta coefficients and statistically significant *p*-values. It was proven from the results that PE, HM, and SI showed favorable substantial impacts on INFC, which supported the hypotheses H1, H3, and H5. Furthermore, the path analysis demonstrated that though EE showed a positive path coefficient (0.097), a non-significant *p*-value of 0.065 was also present. This result indicated that EE had no substantial influence on INFC, which refuted the hypothesis H2. Similarly, PR showed a non-significant *p*-value of 0.392, which indicated that PR had no considerable influence on INFC. In line with the research model, PR should have a negative association with INFC. Given that data analysis illustrated a positive path coefficient value of PR (β-value = 0.008), indicating that hypothesis H4 was not supported. In the second part of the path analysis, Table 5 presents the correlations of FC and INFC with ANFC. The results indicated that both FC (β-value = 0.311, *p*-value = 0.000) and INFC (β-value = 0.457, *p*-value = 0.000) had positive path coefficients and statistically significant *p*-values. Thus, FC and INFC were suggested to have substantial favorable influences on ANFC, which was in line with hypotheses H6 and H7.

**TABLE 5:** Hypothesis testing.

Hypo		Beta	CI-Min	CI-Max	*T*	*p*	*r* ^2^	*f* ^2^	*Q* ^2^	Decision
H1	PE → INFC	0.348	0.224	0.474	4.649	0.000	0.749	0.142	0.587	Supported
H2	EE → INFC	0.097	0.002	0.204	1.516	0.065		0.011		Rejected
H3	HM → INFC	0.251	0.150	0.341	4.250	0.000		0.090		Supported
H4	PR → INFC	0.008	−0.041	0.058	0.274	0.392		0.000		Rejected
H5	SI → INFC	0.298	0.205	0.380	5.829	0.000		0.167		Supported
H6	FC → ANFC	0.311	0.210	0.416	4.847	0.000	0.526	0.079	0.373	Supported
H7	INFC → ANFC	0.457	0.358	0.551	7.671	0.000		0.170		Supported

*PE, performance expectancy; SI, social influence; EE, effort expectancy; FC, facilitating conditions; HM, hedonic motivation; PR, perceived risk; INFC, intention to adopt NFC M-payments; ANFC, adoption of NFC M-payments.*

The coefficient of determination (*r*^2^) assesses the study model predictive power, which demonstrates the number of variances in endogenous constructs that are represented by all the exogenous constructs associated with it (Hair et al., 2017). The values of *r*^2^ that exceed or are equal to 0.75, 0.50, or 0.25 are categorized as significant, moderate, or weak, respectively (Hair et al., 2017). Overall, the components PE, HM, and SI presented a significant proportion (*r*^2^ = 74.9%) of the variation in adoption intention of NFC M-payment. Simultaneously, FC and INFC were able to explain a moderate proportion (*r*^2^ = 52.6%) of the variance in the actual adoption of NFC M-payments. As a predictive accuracy criterion, the *Q*^2^ value should be examined besides the *r*^2^ values (Hair et al., 2017). Moreover, *Q*^2^ values higher than zero in the structural model for a specific reflective endogenous latent variable illustrated the model predictive relevance for the dependent variable (Hair et al., 2017). The values of *Q*^2^ (e.g., 0.587 for INFC and 0.373 for ANFC) presented in Table 5 were found to have values higher than zero, indicating that all of the components had a significant predictive relevance.

### Mediating Effect

The mediating effects of INFC on the relationships between ANFC and PE, EE, HM, PR, and SI are presented in Table 6. The result indicated that INFC positively and significantly mediated the association between ANFC and PE (β-value = 0.159, and *p*-value = 0.000), HM (β-value = 0.115, and *p*-value = 0.000), and SI (β-value = 0.136, and *p*-value = 0.000). Furthermore, the mediation effects of INFC in the association of ANFC and EE had a non-significant *p*-value (0.081), revealing that INFC had no significant mediating impact in the relationship. Simultaneously, mediation analysis of INFC in the association of ANFC and PR resulted in a *p*-value of 0.394, which was larger than 0.05. This result indicated that INFC had no significant mediating influence in the relationship between ANFC and PR.

**TABLE 6:** Mediation effects.

Relations	β	CI-Min	CI-Max	*t-value*	*p*	Decision
PE → INFC → ANFC	0.159	0.105	0.221	4.410	0.000	Supported
EE → INFC → ANFC	0.044	−0.005	0.098	1.403	0.081	Rejected
HM → INFC → ANFC	0.115	0.063	0.160	3.778	0.000	Supported
PR → INFC → ANFC	0.004	−0.017	0.029	0.270	0.394	Rejected
SI → INFC → ANFC	0.136	0.089	0.189	4.494	0.000	Supported

*PE, performance expectancy; SI, social influence; EE, effort expectancy; FC, facilitating conditions; HM, hedonic motivation; PR, perceived risk; INFC, intention to adopt NFC M-payments; ANFC, adoption of NFC M-payments.*

### Importance-Performance Factors

An importance-performance matrix analysis was conducted using the ANFC. All seven constructs aimed to determine the factors that were more significant in the adoption of NFC M-payments consumers. The importance-performance matrix analysis (Table 7) illustrated that even though EE had no significant influence on ANFC, it showed the highest performance value (79.054). The second-highest performance value (77.791) was for PE, followed by FC (73.362), INFC (72.877), HM (71.365), and SI (66.464). For the total effect evaluation, the most crucial factor was INFC with the total effect value of 0.457, followed by FC (0.311), PE (0.159), and SI (0.136).

**TABLE 7:** Importance-performance matrix.

Target construct variables	Adoption of NFC M-payments	
	Total effects	Performance
Performance expectancy	0.159	77.791
Effort expectancy	0.044	79.054
Hedonic motivation	0.115	71.365
Perceived risk	0.004	59.165
Social influence	0.136	66.464
Facilitating conditions	0.311	73.362
Intention to adopt NFC M-payments	0.457	72.877

## Discussion

The study results indicated that PE had a profound effect on INFC, which was in line with the results from the previous NFC M-payments studies conducted by Khalilzadeh et al. (2017); Morosan and DeFranco (2016), Karjaluoto et al. (2020), and Liébana-Cabanillas et al. (2021). The findings indicated that the degree to which NFC M-payments enhanced daily transaction activities had a substantial impact on INFC. This condition could be attributed to a variety of factors, such as the convenience of using it at any time and place (Gupta and Arora, 2019), including the flexibility in being cashless. As a result, NFC M-payments were regarded by the users as a fast and safe method of money transaction, which contributed to the widespread adoption of it.

The study demonstrated a non-significant effect of EE on the INFC, which supported the findings of previous research outcomes on mobile payments (Koenig-Lewis et al., 2015; Morosan and DeFranco, 2016; Oliveira et al., 2016; Lin et al., 2020). The statistically insignificant relationship between EE and INFC suggested that the existing NFC M-payment systems are already enough convenient for users to use. Hence, the perception of the hard effort to perform transaction activities using the technologies is no longer a discernible element. Furthermore, it was highlighted that the influence of EE could diminish with time as users gain proficiency through continuous use of a particular technology (Venkatesh et al., 2003). The majority of young people recognize themselves as highly adept and experienced smartphone users, and they often utilize a range of mobile transaction applications. As a result, it was projected that their perception of the ease of use of NFC M-payments was not a significant factor of adoption intention, providing a plausible explanation for the findings.

In respect of HM, a substantial and favorable influence of it on INFC was recorded, which indicated that the finding was analogous to a few earlier studies on NFC M-payments (Morosan and DeFranco, 2016; Khalilzadeh et al., 2017). However, this result contradicted the majority of previous studies in the mobile payment context (Koenig-Lewis et al., 2015; Gupta and Arora, 2019; Hussain et al., 2019; Karjaluoto et al., 2020; Liébana-Cabanillas et al., 2021). Furthermore, the result illustrated that the young consumers of NFC M-payments were pleased with the use of technology and application of user interface, add-ons, promotional deals, and other real-time features. Additionally, in contrast to the consumers in other countries, the users of NFC M-payments were enthusiastic and satisfied with the use of these services.

Although the study suggested that the PR should have a negative impact on users’ adoption intention of NFC M-payments, the statistical analysis identified a positive path coefficient between PR and INFC. Furthermore, the PR showed no significant impact on the consumers’ inclination to use NFC M-payments. The finding supported the earlier studies of Chawla and Joshi (2019) in the mobile payment context, including Ooi and Tan’s (2016) study in the context of smartphone-based credit-card. In this case, the PR was found to not possess a considerable influence on the users’ behavioral intention to accept the services. In this circumstance, the individuals’ beliefs regarding the use of mobile payments would cause no privacy and/or security risks and increase the importance of their willingness to acquire mobile payment services. However, this study results contradicted the earlier findings by Liébana-Cabanillas et al. (2021); Karjaluoto et al. (2020), and Penney et al. (2021), who found that the PR had a substantial negative impact on the users’ intentions to use mobile payments. The possible interpretation of this contradiction could be that the NFC-based service providers are maintaining a certain high degree of security and hacker-free services. Users show high reliance on the services and do not have a strong concern about any vulnerabilities or risks associated with the use of these services.

The influence of SI on the INFC was found to be substantially positive, which was in line with the findings from prior research works in the context of NFC M-payments conducted by Khalilzadeh et al. (2017); Morosan and DeFranco (2016), and Liébana-Cabanillas et al. (2021). It could be argued that the consumers’ opinions and intentions regarding the acquisition of NFC M-payments are heavily influenced by the suggestions and recommendations of popular and prominent individuals in society. Additionally, consumers could be encouraged and convinced by their close friends, family, relatives, and peers to adopt NFC M-payments.

The study analysis demonstrated a profound impact of FC on the ANFC, which was in line with earlier studies that focused on FC as an influencing factor in mobile payment adoption (Morosan and DeFranco, 2016; Alalwan et al., 2017; Chawla and Joshi, 2019; Lin et al., 2020; Yang et al., 2021). The interpretation highlighted that the users were conscious of the availability of facilities, resources, support, and guidelines from the service providers for the effective and successful acquisition of NFC M-payments. Furthermore, the NFC M-payments were more widely adopted by consumers due to their ability to access real-time information and support, all financial and banking resources, and well-integrated technological resources (e.g., software, hardware, and stable service infrastructure) that could guarantee all facilitating features.

In line with the earlier studies (Oliveira et al., 2016; Alalwan et al., 2017), it was found that the users’ adoption intention of NFC M-payments was a strong determinant of the actual adoption of the services among youth. This result indicated that the users of NFC M-payments had a higher likeliness to use the service when they had the intention for it. Following the examination of the mediating impact of INFC, a substantial mediating effect of INFC was found in the associations between PE and ANFC, HM and ANFC, SI, and ANFC. The previous research findings by Koenig-Lewis et al. (2015); Gupta and Arora (2019), Yang et al. (2021), and Karjaluoto et al. (2020) showed partially similar results. It could be concluded from the analysis that PE, HM, and SI had a substantial influence on rapid actual adoption of NFC M-payments among youths when users showed a high level of intention to use these services.

## Implication

### Theoretical Implication

This study developed a sophisticated framework that extended on the well-known theoretical model (e.g., UTAUT2) to further analyse other crucial components and constraints of NFC M-payments adoption, including the possibility for consumers to respond to those factors. Based on the statistical analysis, the proposed model showed a high level of explanatory power in terms of forecasting customer adoption intention and actual use of NFC M-payments. The study conceptual framework has theoretically augmented the UTUAT2 model by incorporating an important factor, such as the PR, which was found to be rarely investigated in the earlier studies of NFC M-payments adoption in Malaysia. Moreover, the impact of PR was found to contradict the previous findings on contactless payment adoption by consumers from other countries. This factor could be considered an important contribution of this study to the existing literature, which created opportunities for further investigation in other circumstances.

### Managerial Implication

The primary goal of this study is to determine and validate several components that strongly influenced behavioral intention and the actual adoption of NFC M-payments among young users. Given the strong influence of PE on INFC, service providers should work on improving the functionality and efficiency of the services to ensure that the M-payment services could be adopted and used more productively to fulfill the consumer-specific purposes. Financial corporates should launch additional promotional efforts advertising NFC M-payments, highlighting their effectiveness and convenience of use (e.g., faster and simpler purchasing, higher productivity, improved transaction performance, and secure transactions at any time and place) and could foster the users’ attention. Given that SI and HM were two crucial drivers for NFC M-payments adoption among users, service providers should consider various methods to increase the appeal of their services to consumers through the addition of sophisticated add-ons, entertaining features, and freebies with promotional deals to encourage customers to use their services more frequently and generate positive word-of-mouth. Moreover, marketers should consider appointing popular social influencers to campaign the compelling advantages of using the services to boost popularity. Service providers should identify the early adopters and encourage the use of NFC M-payments as they may serve as references in the future to enable more widespread adoption. Marketers should focus on the social media marketing methods to promote the word-of-mouth and favorable recommendations since the social media networks have improved this interactions between users. Based on the study’s emphasis on FC as a strong driver of actual acquisition of NFC M-payments, providers should deploy an advanced machine learning algorithm to simplify transaction procedures and make facilitating features easily accessible to consumers (e.g., strong network coverage, platform-independent application, coverage for all business sectors, increasing merchants, partnering all commercial banks, and financial institutes among others). Although PR was reported to be a non-significant factor in users’ adoption of NFC M-payments, this study emphasized the significance of concentrating and extending certified resources to develop a more secure system infrastructure and environment. Because consumers are worried about their privacy and personal information, mobile payment providers must use enhanced encryption tools to support transactions more securely (Flavián et al., 2020). Furthermore, in the event of scamming assaults, a money-back guarantee approach might be another successful marketing strategy that would be more advantageous in resolving risk barriers. Service providers should also assure the availability of on-demand and on-time expert assistance for any concerns that may occur upon the use of the system.

## Conclusion

The NFC M-payment technology is the fastest expanding area in financial transaction technology, which strengthens the interest among scholars to investigate its adoption behavior among consumers; particularly, when the significant constraints are taken into account. Addressing Malaysian customers’ lower adoption rate of NFC M-payments, this study highlighted the importance of examining the core elements that could influence the consumers’ intention to use NFC M-payments. This research adopted the UTAUT2 as the underpinning theoretical model and augmented it with an essential component (e.g., PR) to predict the users’ intention to use and the actual adoption behavior for NFC M-payments. It was found from the analysis that PE, HM, and SI were strong predictors of consumers’ intent to use NFC M-payments. Furthermore, both INFC and FC were found to be the influencing factors in determining the actual usage of NFC M-payments. Concurrently, INFC substantially mediated the relationships between PE, HM, SI, and actual usage behavior. On the other hand, the study analysis presented no statistically significant influence of EE and PR on the intention to use NFC M-payments among youths. Similarly, no substitute was present for recognizing the essential aspects of users’ intention to design, refine, and deploy the NFC M-payments services, which could contribute to high user satisfaction and enhancement enthusiasm among consumers to use this financial transaction technology. Overall, the results would assist all stakeholders, specifically service providers, merchants, marketers, and policymakers, in the development of strategies to increase the widespread acquisition of NFC M-payments. Additionally, the study was expected to create opportunities for future research work to be performed in this area.

### Limitations and Recommendations for Future Research

Although this study represented a successful endeavor in the domain of NFC M-payments acceptance and use, several constraints were also present. First, the study used a limited sample of 370 Malaysian young users of NFC M-payments, which restricted the generalizability of the findings. The demographic characterization of the study dataset also revealed that the majority of respondents were young in age and well-educated, and considered to have received adequate technology and Internet knowledge and skills. As a result, several concerns were present regarding the application of the results to other groups of the contemporary population with distinct demographic features (e.g., income, age, gender, education level, and technology experience). Therefore, it was suggested that future research extends the findings of this study by including an analogous research model with a larger sample size focusing on various demographic populations from the cross-country perspective on the distinct cultural, environmental, economic, and technical contexts. This action could be made by investigating the more determinants of behavioral intents of adopting NFC M-payments. Furthermore, the current study findings were based on the cross-sectional data, which raised concerns regarding the long-term relevance of these findings. This issue might be attributed to the fact that customers’ attitudes, perceptions, and awareness of these types of technologies have a higher possibility to transform over time. Finally, further longitudinal studies are hoped to create a better understanding of the issues and the solutions to stabilize or modify the influence of the elements over time.

## Data Availability Statement

The original contributions presented in the study are included in the article/supplementary material, further inquiries can be directed to the corresponding author.

## Ethics Statement

Ethical review and approval was not required for the study on human participants in accordance with the local legislation and institutional requirements. The patients/participants provided their written informed consent to participate in this study.

## Author Contributions

CM, SJ, and TA: conceptualization, methodology, analysis, and writing – original draft. AA and FN: methodology, formal analysis, and writing – review and revision. All authors contributed to the article and approved the submitted version.

## Conflict of Interest

The authors declare that the research was conducted in the absence of any commercial or financial relationships that could be construed as a potential conflict of interest.

## Publisher’s Note

All claims expressed in this article are solely those of the authors and do not necessarily represent those of their affiliated organizations, or those of the publisher, the editors and the reviewers. Any product that may be evaluated in this article, or claim that may be made by its manufacturer, is not guaranteed or endorsed by the publisher.

## Footnotes

1
https://webpower.psychstat.org/wiki/tools/index
